# Prevalence and Risk Factors of Unintentional In-Home Injuries in Older Adults

**DOI:** 10.3390/medicina61071235

**Published:** 2025-07-08

**Authors:** Ok-Hee Cho, Hyekyung Kim

**Affiliations:** 1 Department of Nursing, College of Nursing and Health, Kongju National University, Gongju 32588, Republic of Korea; ohcho@kongju.ac.kr; 2 Department of Nursing, Catholic Kwandong University, Gangneung 25601, Republic of Korea

**Keywords:** aged, injury, prevention, home environment

## Abstract

*Background and Objectives*: Older adults are a vulnerable population to unintentional injuries due to age-related physiological decline and the presence of various chronic conditions. Unintentional injuries occurring in the home, such as falls, burns, poisoning, cuts, and suffocation, have been reported at higher rates in this age group compared to younger populations. This study examines the prevalence and types of unintentional in-home injuries in older adults and identifies the risk factors associated with falls and cuts/collisions. *Materials and Methods*: A cross-sectional study was conducted on 309 older adults (aged ≥ 65 years) recruited from eight senior welfare centers in South Korea. *Results*: The most frequent cause of injury was falls (28.7%), followed by cuts/collisions (27.0%), burns/fire (11.4%), and other injuries (8.1%). In the model adjusted for age and sex, risk factors for falls included a history of outdoor falls or indoor cuts/collisions, dizziness, and the use of two or more medications. Risk factors for cut/collision injuries included a history of indoor burns or falls, numbness in hands and feet, and visual impairment. *Conclusions:* To effectively prevent home injuries among older adults, it is crucial to focus not only on falls but also on frequent minor injuries caused by cuts and collisions.

## 1. Introduction

Unintentional injuries among older adults are a significant public health concern and a leading cause of morbidity and mortality [1,2]. Older adults are particularly vulnerable due to physiological decline associated with aging as well as an increased risk of various health conditions such as stroke, dementia, cardiovascular disease, and diabetes [3]. Unlike younger individuals, the older population is more susceptible to severe injuries even from low-impact incidents [4,5]. In the United States (U.S.), the mortality rate from unintentional injuries among adults aged 65–84 was 66.87 per 100,000 between 1999 and 2012, nearly ten times higher than that of children aged 0–14 [6]. In South Korea, one of the world’s fastest-aging populations, the injury-related mortality rate among women aged 80 and older doubled between 1996 and 2006 [7]. As of 2021, the injury-related mortality rate among men aged 70 and older was 15 times higher than that of men aged 20–29 [8]. Consequently, many countries are developing policies designed to reduce preventable injuries and promote safe aging, prioritizing the prevention of unintentional injuries [9].

While home is generally perceived as a haven and is often overlooked as a potential site for injuries, it can present significant risks. In-home injuries include those that occur within the home and its immediate surroundings [10]. These injuries particularly affect individuals who spend extended periods at home and have limited self-protective capabilities, including infants, children, women, older adults, individuals with poor health, and socioeconomically disadvantaged groups [11]. Risk factors for in-home injuries include advanced age (≥65 years), young age (<15 years), female sex, prolonged time spent on household tasks, smoking, dissatisfaction with health, low income, living alone, and having a garden [4,10,11]. While most household injuries are minor, such as bruises or superficial wounds, and require minimal medical intervention, some can be severe [10,12]. Sikron et al. [13] reported that among 26,921 patients admitted to eight trauma centers for household injuries, 37% were older adults (≥65 years), 60% of whom required surgery. Injuries among older adults can lead to health complications, functional limitations, and loss of independence, sometimes necessitating admission to long-term care facilities [14]. However, the clinical outcomes of these injuries vary depending on the injury type, with injury risk influenced by the complexity of contributing factors and preventive behaviors [15,16].

The increasing number of emergency department visits and hospitalizations due to trauma in older adults also imposes significant strain on healthcare systems and contributes to the societal economic burden [17]. While childproofing homes is common, implementing home safety measures for older adults is not as widespread [10,18]. Therefore, developing and implementing preventive strategies to minimize unintentional in-home injuries among older adults is crucial [15].

Falls are the most common cause of in-home injuries among older adults [4,13,19], involving multiple risk factors: aging, sex, history of falls, health issues related to chronic diseases and polypharmacy, vision impairment, cognitive and neurological disorders, impaired balance and gait abilities, and underweight [5,20]. Additionally, psychological factors such as depression and fear of falling, as well as environmental factors such as uneven flooring, poor lighting, and unsafe home structures, further increase the risk of falls [21,22]. Moreover, injuries related to burns, poisoning, cuts, and suffocation are more prevalent among older adults than younger age groups [10]. While falls have been extensively studied, in-home injuries caused by cuts, knocks, and collisions—common among older adults and second only to falls [12,23,24]—have received comparatively less research attention. Additionally, studies on the interrelationships among different in-home injury types are scarce.

Although findings based on hospital data provide reliable insights, they may overlook less severe incidents and offer only a partial understanding of the specific circumstances and causes of injury. In contrast, qualitative studies provide in-depth insights into individual injury experiences but lack generalizability.

To address these limitations, this study utilized a structured questionnaire and individual interviews to examine the frequency and specific causes of unintentional in-home injuries across eight injury types among older adults over the past year. The study sought to identify the risk factors for the most common injury types: falls and cuts/collisions. Investigating unintentional in-home injuries, experiences with different injury types, and associated risk factors is essential in developing appropriate prevention strategies and ensuring safer living environments for older adults.

## 2. Materials and Methods

### 2.1. Participants

Employing a cross-sectional design, participants were recruited from older adults visiting eight senior welfare centers in South Korea. Senior welfare centers were selected as recruitment sites to ensure a diverse age range among community-dwelling older adults, as they serve as primary venues for engaging in various leisure and cultural activities. Surveys were administered to individuals who voluntarily agreed to participate and met the inclusion criteria: (1) aged 65 years or older, (2) residing in a private home, and (3) able to understand and respond to the questionnaire independently. Individuals diagnosed with mental illness, dementia, or those unable to communicate effectively were excluded. A minimum sample size of 308 participants was calculated using G*Power 3.1.9. (significance level = 0.05, power = 0.80, odds ratio = 1.5). To account for potential dropouts and incomplete responses, 350 surveys were distributed, of which 317 were returned (90.6%). After excluding eight incomplete surveys, 309 responses (88.3%) were included in the final analysis.

This study was approved by the Institutional Review Board (IRB) (No. KNU_2023-85). Participants were informed about the study procedures, anonymity, and data confidentiality before providing written informed consent.

### 2.2. Survey Instrument

The survey instrument comprised two main sections: (1) sociodemographic and risk factor information and (2) injury types and causes. The survey was developed by the research team and validated by a six-member expert panel (one physician, two nurses, one gerontology professor, and one occupational therapist). Additionally, a pilot survey was conducted with five older adults who met the inclusion criteria to assess item validity and readability.

The first section comprised two subsections: (1) eleven items assessing sociodemographic characteristics, including age, sex, education level, marital status, living arrangement, housing type and status, religious affiliation, employment status, economic status, and history of falls in the past year, and (2) eight items assessing injury-related risk factors, including the number of chronic diseases, number of medications, self-rated health status (good/average/poor), sleep satisfaction (satisfied/average/dissatisfied), frailty level (robust/prefrail/frail), perceived physical function decline (vision impairment, hearing impairment, numbness in hands and feet, tremors in hands and feet, and dizziness), use of mobility aids, and depression.

Frailty was measured using the K-FRAIL, the Korean version of the FRAIL scale [25], a questionnaire comprising five items (fatigue, resistance, ambulation, illness, and weight loss) that screen frailty status without requiring a physical examination. A higher score indicates a higher level of frailty (0 = robust, 1–2 = prefrail, 3–5 = frail). The perceived physical function decline was assessed based on the response to the following question: “Does a decline in any of the following physical symptoms (vision impairment, hearing impairment, hands/feet numbness, hands/feet tremors, dizziness) cause difficulty in your daily life?” (yes/no binary response option). Depression was measured using the Korean version of the PHQ-2 [26]. This two-item depression screening tool is rated on a four-point scale, with a total score ranging from 0 to 6. A score of 3 or higher is considered a positive screening result, indicating the presence of depressive symptoms.

The second section assessed injury-related risk factors based on previous studies [14,15] and included eight common in-home injury types: falls, cuts/collisions, burns/fire, suffocation/swallowing disorders, medication-related incidents, dehydration, heat-related illnesses, and cold-related illnesses. Among these, falls refer to experiences such as tripping, slipping, or collapsing; cuts involve being scratched, cut, or stabbed by objects such as knives or glassware; and collisions indicate incidents such as bumping into furniture or walls, being struck by a person, or being hit by a heavy object. Participants were asked about their injury history related to any of these causes in the past year, including occurrence (yes/no), frequency of occurrences, and whether hospital treatment was required (yes/no).

### 2.3. Data Collection

Data were collected from September to November 2023. Two researchers and two trained surveyors visited the selected senior welfare centers after explaining the purpose of the study and obtaining cooperation from the center heads. On the survey day, participants were informed about the study’s objectives and procedures. Those who voluntarily provided written consent were administered the questionnaire. The researchers and surveyors read the questions aloud and recorded participants’ responses. Each interview took approximately 20 min to complete.

### 2.4. Data Analysis

Data were analyzed using SPSS version 27.0. Descriptive statistics were used to summarize participants’ sociodemographic characteristics, injury types, and treatments received. Multiple logistic regression, adjusted for age and sex, was used to identify risk factors for fall and cut/collision injuries. The results are presented as odds ratios (ORs) with 95% confidence intervals (CIs).

## 3. Results

### 3.1. Participant Characteristics

The participants’ mean age was 76.8 years (range: 65–96 years), with those in their 70s accounting for the highest proportion (n = 140, 45.3%). Most were female (n = 199, 64.4%), had elementary school education (n = 87, 28.2%), and were married (n = 189, 61.2%), with 150 (48.5%) living with their spouse only. Additionally, 148 (47.9%) lived in detached houses, 175 (56.6%) did not own their residence, 198 (64.1%) identified with a religion, 217 (70.2%) were unemployed, and 229 (74.1%) considered their economic status to be “middle.” Within the last year, 104 participants (33.7%) experienced a fall, with 87 (83.7%) falling indoors, 53 (51.0%) outdoors, and 36 (34.6%) in indoor and outdoor settings (Table 1).

### 3.2. Prevalence of In-Home Injuries and Treatment Experience

Among the participants, 139 (45.0%) experienced at least one in-home injury in the past year. Of those injured, more than half (84, 27.2%) sustained injuries of two or more types, while 55 (17.8%) experienced only one type of injury. The most common injuries were falls (28.7%), cuts/collisions (27.0%), burns/fire (11.4%), and other injuries (8.1%).

Of the 87 participants (28.2%) who experienced falls at home, 42.5% sought hospital treatment. The most frequently reported causes of falls were slipping on wet bathroom floors, slipping suddenly due to loss of strength in an obstacle-free area, and tripping over objects on the floor. Meanwhile, of the 83 participants (27.0%) who experienced cuts or collisions, 8.4% sought hospital treatment. The most common cause was cuts from sharp objects. In contrast, of the 35 participants (11.4%) who reported burns or fire, 6 (17.1%) required hospital treatment. Other injuries were reported by 25 participants (8.1%), with 8 (32.0%) requiring hospital treatment (Table 2).

### 3.3. Risk Factors for Falls and Cuts/Collisions

Multiple logistic regression analysis, adjusted for age and sex, identified the following risk factors for falls: taking two or more medications compared to no medication (OR = 3.59, 95% CI = 1.06–12.18), “average” health compared to “unhealthy” (OR = 0.43, 95% CI = 0.19–0.99), perceived vision impairment (OR = 0.44, 95% CI = 0.21–0.92), dizziness (OR = 4.37, 95% CI = 1.90–10.05), history of outdoor falls (OR = 8.36, 95% CI = 3.52–19.82), and history of indoor cuts/collisions (OR = 6.20, 95% CI = 2.85–13.48). The risk factors for cut/collision injuries included “frail” (K-FRAIL scores ≥ 3) compared to “robust” (OR = 0.16, 95% CI = 0.04–0.67), perceived vision impairment (OR = 2.15, 95% CI = 1.07–4.33), perceived hearing impairment (OR = 0.47, 95% CI = 0.22–0.99), numbness in hands and feet (OR = 3.13, 95% CI = 1.39–7.07), history of indoor falls (OR = 6.29, 95% CI = 3.03–13.02), and history of indoor burns (OR = 8.50, 95% CI = 3.31–21.85) (Table 3).

## 4. Discussion

This study examines the prevalence of eight common types of in-home injuries and identifies risk factors associated with falls and cuts/collisions. Additionally, it explores the frequency and causes of other types of in-home injuries. This study differentiates itself from the existing literature in three ways. First, it examines not only falls, the most prevalent in-home injury, but also other injury types and their specific causes. Second, it analyzes the risk factors for falls and cuts/collisions injuries while considering eight common in-home injury types. Third, it includes minor injuries that do not require medical attention. The findings of this study contribute to the body of literature supporting the association between unintentional in-home injuries among older adults and their risk factors, reinforcing the notion that such injuries can be prevented. They may also assist in developing intervention programs aimed at educating older adults on protective measures and improving their quality of life.

Among the study participants, 45.0% had experienced an in-home injury, a rate comparable to findings from large-scale studies that reported prevalences of 40.6–42% [16,18]. Additionally, in a U.S. study, over half of the approximately 10.2 million injuries that occurred in domestic settings in 2006 took place within homes and adjacent areas [27]. Similarly, according to South Korea’s national statistics, 54.6% of injuries in 2021 occurred in homes and residential areas [8], aligning with the findings of this study.

In this study, 27.2% of participants experienced at least two different types of in-home injuries. Similarly, Zhang et al. [16] reported that among 3752 community-dwelling older adults in China, 59.8% experienced one in-home injury, 15.8% experienced two, and 23.7% experienced three or more [19]. Collectively, these findings support the notion that even a single fall can predict recurrent falls [28]. These findings further suggest that exposure to common risk factors increases the risk of multiple injuries. A study conducted in New Zealand [29] estimated that an additional home hazard increased the probability of an associated injury by 22% and that the risk of medically significant injuries increased with the number of household hazards. Identifying common hazards leading to injury can thus be instrumental in quantifying home injury risks and developing assessment tools to prioritize hazard mitigation efforts.

In this study, the most common types of in-home injuries were falls, cuts/collisions, burns/fire, and other injuries, a pattern consistent with previous research [24]. Notably, injuries from causes other than falls accounted for over 70% of the cases, highlighting their significance and the need for greater attention. The prevalence of in-home falls was 28.2%, lower than the 53.1% reported in a study [19] on rural older adults in Southern Karnataka, India, but comparable to the 20–30% rates reported in multiple countries, irrespective of location [30,31,32,33]. Among those who experienced a fall, 42.5% sought hospital treatment, aligning with findings from U.S. studies conducted between 2012 and 2018, where approximately 37% of older adults who had fallen required medical attention or experienced at least one day of activity limitation [33,34]. These statistics also indicate that more than 50% of the participants who experienced a fall did not seek medical intervention and managed the injury at home. Non-fatal injuries also pose a potential risk, as they may contribute to other injuries by exacerbating underlying vulnerabilities [10]. However, whether an individual seeks medical treatment is often used as a proxy indicator of injury severity, which may not accurately reflect the functional impact of the injury. The absence of medical care does not necessarily indicate the absence of functionally significant harm. This approach may underestimate the cumulative effects of minor but recurrent injuries. Therefore, future studies should incorporate more precise indicators and methods to assess the functional or clinical severity of injuries.

While the prevalence of cuts/collisions (27.0%) was comparable to falls, only 8.4% of those injured sought hospital treatment. Hence, although the number of cases was as high as that of falls, the proportion requiring medical attention was significantly lower. This suggests that cuts/collisions may be underreported due to recall bias or a tendency to perceive them as minor injuries. To gain a comprehensive understanding of in-home injuries, it is essential to consider severe injuries requiring hospital treatment and less severe injuries that can be managed without medical interventions.

The most frequently reported causes of falls in this study included slipping on wet bathroom floors and tripping over objects on the floor. These modifiable and predictable environmental factors can be effectively prevented by raising awareness of fall risks [31] and implementing measures to modify environmental factors [22], such as installing grab bars and non-slip mats in bathrooms. However, a notable proportion of falls were reported as occurring in obstacle-free areas due to a sudden loss of strength, a pattern that differs from falls among younger and middle-aged adults [5]. While the cause of sudden weakness cannot be definitively ascribed to symptoms of muscle weakness, chronic diseases, and dizziness, this finding highlights the need to consider these symptoms and their complex interactions with other risk factors. It also suggests that fall prevention strategies based solely on environmental modifications may not be sufficient [35]. Joseph et al. [35] reported that most older adults who had fallen experienced subjective premonitory feelings before the fall, such as weakness of limbs (41.8%), giddiness (15.6%), vertigo (5.7%), and dimness (5.7%). These findings highlight the need for an injury surveillance system that offers detailed data on in-home injuries among older adults.

The most frequently reported causes of cuts or collisions in this study were scratches or cuts from sharp objects and collisions with household furniture or walls. These findings may be attributed to the increasing number of older couples and adults living alone, requiring them to perform more cooking and household tasks independently [16]. Additionally, reduced agility and balance impairments while performing activities of daily living and moving within the home may contribute to these injuries [4]. A previous study [16] also found that older adults aged 65–74 who were more active at home were more likely to sustain cuts, indirectly supporting the findings of this study.

After adjusting for age and sex, multiple regression analysis was conducted, incorporating risk factors across the eight injury types. Older adults who had previously experienced a fall outdoors were eight times more likely to experience a fall indoors compared to those with no history of outdoor falls. Falls indoors and outdoors may be influenced by different risk factors, such as environmental conditions, lifestyle, and activity range, which vary by age and sex [20]. While this study did not assess whether a participant’s first fall occurred indoors or outdoors, 51% of falls occurred outdoors, and these individuals had a high risk of indoor falls.

Older adults with a history of cut/collision injuries were also six times more likely to experience falls, and those with a history of falls were six times more likely to experience cut/collision injuries.

Although the temporal sequence of fall and cut/collision injuries was not assessed, the findings suggest a potential association between these two injury types. However, as the study is based on cross-sectional data, caution is warranted in interpreting the results, as temporal or causal relationships cannot be inferred. A systematic review [36] on fall risk factors in older adults described cuts and collisions at home as warning signs of a fall. Recognizing the interconnectivity between different injury types and responding proactively to early warning signs could be an effective prevention strategy to minimize further injuries.

Dizziness is one of the most frequently reported symptoms among older adults with a history of falls and is therefore considered a major risk factor for falls. It is a symptom that may be associated with stroke, hypertension, and chronic respiratory diseases [37,38], and older adults are at greater risk due to their reduced ability to recover balance quickly [39]. Specifically, orthostatic hypotension-related dizziness increases fall risk by 2.5 times [40], and older adults who experience dizziness are 2.29 times more likely to fall [35]. Polypharmacy is also a well-documented risk factor for falls [15,28], as uncontrolled medical conditions and nonadherence to prescribed medications can impair attention, judgment, and coordination, increasing fall risk [35]. Regular monitoring, systematic screening, and proper management of inappropriate medications can help reduce the risk of falls among older adults.

In this study, a positive perception of health status was associated with a lower risk of falls, aligning with previous research [41] and suggesting that older adults with a higher perceived health status tend to have greater fall efficacy, reducing fall risk. Similarly, Grundstrom et al. [34] found that among adults aged 85 and older, those who rated their health as poor had a higher fall risk compared to younger older adults, whereas those who perceived themselves as healthy had a fall risk similar to younger older adults, supporting the findings of this study. Meanwhile, although regular physical activity is known to be an important factor in reducing fall risk among older adults [31], this study did not assess the actual levels of physical activity or exercise, despite considering the perceived health status. Therefore, future studies should take into account the actual level of physical activity, which may serve as a valuable foundation for developing more effective fall prevention strategies.

Visual impairment was identified as a protective factor against falls, contradicting previous research that reported it as a risk factor [5]. This paradoxical finding may be explained by the increased fear of falling among individuals with poor vision, leading them to restrict their activities, reducing their potential for falls [42]. However, this interpretation is speculative and should be approached with caution to avoid overinterpretation. Future research should incorporate both subjective and objective assessments of visual function to enable a more precise analysis. In addition, follow-up studies are needed to better understand the discrepancy between the findings of this study and those reported in previous research. While the importance of fall prevention interventions in hospital settings has been widely documented, fall prevention in home environments remains understudied. Although community-dwelling older adults may not experience the same acute health conditions as their hospitalized counterparts, they remain highly susceptible to falls due to chronic diseases, declining health status, and impaired physical function, necessitating individual awareness and societal vigilance. Fortunately, these risk factors can be addressed through exercise, vision assessment and correction, environmental modifications, and medication management. However, while many older adults can recognize certain fall risk factors and understand the consequences of falling, some underestimate their own risk, leading to a lack of engagement in preventive measures [43]. Therefore, enhancing risk awareness should go hand in hand with strengthening proactive fall prevention behaviors to ensure effective injury prevention.

Individuals with a history of burns, previous indoor falls, numbness in hands and feet, or vision impairment were found to be at an increased risk of cuts and collisions. Ferrante [24] reported that cut injuries in women at home are primarily linked to knife use in kitchens, while in men, they are associated with tool use. Burns/fire were the third most common type of in-home injury after falls and cuts/collisions. Given that kitchen environments involve the simultaneous use of knives and fire, the risk factors for burns/fire and cuts/collisions are likely interconnected. A study on older adults in rural China [16] indirectly supports this finding, reporting that firewood use for heating and cooking exposed them to increased risks of burns and knife-related injuries. Based on the finding that individuals with a history of indoor falls were six times more likely to experience cuts/collisions, it can be assumed that these injuries are interrelated, with one potentially leading to the other.

This study also identified numbness in hands and feet and vision impairment as risk factors for cuts/collisions. Previous research [16] found that older adults with diabetes, arthritis, and cataracts had 42%, 27%, and 38% higher injury rates, respectively, indirectly supporting these findings. Reduced sensation in the hands and feet can increase injury risk by delaying recognition of an injury, impairing tactile feedback, and slowing reaction time. Decreased spatial awareness, slower reflexes, and temperature sensitivity can make it more challenging for older adults to respond quickly to potential hazards. Interestingly, vision impairment was simultaneously identified as a risk factor for cuts/collisions and a protective factor against falls. Since vision deteriorates with age, cuts/collisions may be more prevalent among older adults who remain active in household activities despite declining vision [20]. Older adults are also more likely to experience burns and cuts due to age-related physical changes, comorbidities, and financial limitations [16]. Despite the availability of various assistive safety devices, lack of information, cost concerns, and personal preferences often lead older adults to continue using traditional methods for handling knives and fire while remaining passive about home modifications [44]. Therefore, health and safety professionals should consider a multifaceted approach to injury prevention, incorporating assistive device education, strength and balance training, home modifications, routine medical check-ups, early screening, safety behavior education, and lifestyle interventions to reduce injury risks among older adults.

Perceived frailty and hearing impairment were identified as protective factors against cuts/collisions, which contrasts with previous research suggesting that functional impairment and reduced independence increase the risk of unintentional injuries in older adults [15]. This discrepancy may be attributed to selection bias, as frail and hard-of-hearing individuals are less likely to engage in cooking, ambulation, and daily activities, which inherently reduces their risk of injury. This finding aligns with previous research indicating that while older adults in poor health may experience more severe injury outcomes, those who are healthier and more active tend to have a higher prevalence of home injuries [5]. Malnutrition among frail older adults may increase their susceptibility to injuries [15], and hearing impairment may further heighten injury risk due to the diminished ability to detect alarm signals and other auditory warnings. It is essential to ensure that these vulnerable older adults are not overlooked when implementing home safety measures.

Aging-related declines in physical function must be considered in the broader context of an individual’s functional capacity, but aging alone does not fully explain the risk factors for in-home injuries. Healthcare providers and caregivers maintain that injuries among older adults can be prevented, whereas some older adults view injuries as an unavoidable aspect of aging and believe that prevention measures are limited in effectiveness. The limited observable impact of prevention strategies suggests that greater efforts should be made to bridge this gap. This study revealed that unintentional injuries among older adults were associated with a history of other injury types, sensory impairments, self-rated health status, and polypharmacy. These findings suggest that in-home injuries are more prevalent among vulnerable groups under specific conditions, reinforcing the notion that modifying these conditions can help prevent injuries. Therefore, enhancing older adults’ awareness through education and providing targeted individual and community-level support are necessary for effective injury prevention in domestic settings. Therefore, concrete and individualized policy interventions are needed to effectively prevent in-home injuries among older adults. These may include educational programs to improve awareness, home modification initiatives to eliminate fall hazards, medication review services to reduce adverse effects, and community-based safety education tailored to older adults. Such a multidimensional approach can enhance the safety of older adults and ultimately serve as a foundation for supporting healthy aging in an increasingly aging society.

### Limitations

First, since data collection on injury occurrence and treatment was self-reported by the participants, recall bias and subjective memory limitations cannot be ruled out. To minimize these biases and limitations, participants were asked detailed questions about injury causes, and a list of injury types was read aloud to facilitate recall. This approach may have helped participants remember more injuries, but minor injuries, which are generally forgotten more easily, may still have been underreported. Second, this study could not determine the temporal sequence or causal relationships between injury occurrences, a limitation of the cross-sectional study design. Future research should employ more multidimensional analyses and longitudinal study designs. Third, because structured questionnaires were used, the study had limited capacity to assess the actual environmental risk factors and available resources in older adults’ homes. Fourth, this study targeted community-dwelling older adults who were healthy and cognitively intact and who utilized senior welfare centers, excluding those who were hospitalized or had major comorbidities. Compared to non-participants, the study participants may have been more physically and socially active and of higher socioeconomic status. As a result, the burden and risk factors of unintentional injuries observed in this study may differ from those in the general older adult population, limiting the generalizability of the findings. Fifth, as this study was conducted in a specific region of South Korea, findings may not be generalizable to older adults from different cultural or demographic backgrounds, and a certain degree of selection bias may exist.

## 5. Conclusions

Approximately half of the older adults surveyed in this study had experienced unintentional in-home injuries, with falls and cuts/collisions being the most common. Multiple logistic regression analyses revealed that a higher risk of these injuries was associated with a history of other injury types, health status, and sensory impairments. Based on these findings, injury prevention efforts should address falls as well as frequent and seemingly minor injuries caused by cuts and collisions. Given the interrelationship between falls and cuts/collisions, implementing regular screening and proactive interventions for high-risk individuals may help prevent more severe injuries. This study contributes to the literature by comprehensively examining the prevalence and risk factors of unintentional in-home injuries among older adults. The findings were derived from direct interviews with community-dwelling older adults, offering insights that are difficult to capture through statistical injury reports alone. These findings will serve as valuable foundational data for developing policies and intervention programs to prevent unintentional in-home injuries in community-dwelling older adults.

## Figures and Tables

**Table 1:** Sociodemographic characteristics (n = 309).

Characteristics	Categories	N (%) or M ± SD
Age (years)		76.8 ± 6.9 (65–96)
	65–70	68 (22.0)
	71–80	140 (45.3)
	≥80	101 (32.7)
Sex	Male	110 (35.6)
	Female	199 (64.4)
Education	None	35 (11.3)
	Elementary school	87 (28.2)
	Middle School	70 (22.7)
	High School	82 (26.5)
	University	35 (11.3)
Marital status	Married	189 (61.2)
	Unmarried	120 (38.8)
Living arrangement	Alone	102 (33.0)
	with spouse	150 (48.5)
	with others	57 (18.5)
Housing type	Apartment	134 (43.4)
	Detached house	148 (47.9)
	Townhouse	27 (8.7)
Housing status	Owner-occupied	134 (43.4)
	Rent	148 (47.9)
	Public rental	27 (8.7)
Religion	Yes	198 (64.1)
	No	111 (35.9)
Employment	Yes	92 (29.8)
	No	217 (70.2)
Economic status	High	14 (4.5)
	Middle	229 (74.1)
	Low	66 (21.4)
History of falls in the past year (n = 104, 33.7%)	Indoors	87 (83.7)
	Outdoors	53 (51.0)
	Both Indoors and Outdoors	36 (34.6)

**Table 2:** Frequency and detailed causes of injuries in the household (n = 309) ^a^.

Injury Type	Occurrence	Frequency of Occurrences	Total Occurrences	Hospitalized ^b^
	N (%)	Min–Max	N	N (%)
Injury Frequency (Past Year)				
None	170 (55.0)			
1 Factor	55 (17.8)			
2 Factors	30 (9.7)			
≥3 Factors	54 (17.5)			
Factor 1: Fall	87 (28.2)	1–22	211	37 (42.5)
Slipping on wet floors (e.g., in bathrooms)	37 (12.0)	1–5	48	18 (48.6)
Suddenly slipping due to weakness in an obstacle-free area	32 (10.4)	1–10	55	13 (40.6)
Falling from furniture (e.g., bed, chair, desk, dining table)	17 (5.5)	1–5	21	6 (35.3)
Falling down stairs	10 (3.2)	1–4	14	5 (50.0)
Tripping over objects on the floor	31 (10.0)	1–10	46	4 (12.9)
Tripping over a door threshold	16 (5.2)	1–10	27	1 (6.3)
Factor 2: Cut and collision	83 (27.0)	1–14	190	7 (8.4)
Scratched or cut by sharp tools (e.g., knife)	47 (15.2)	1–4	70	4 (8.5)
Scratched or cut by glass shards	15 (4.9)	1–4	19	1 (6.7)
Punctured by glass shards	12 (3.9)	1–2	13	1 (8.3)
Collision with furniture or walls	32 (10.4)	1–10	61	-
Injured while moving heavy objects	14 (4.6)	1–5	18	1 (7.1)
Injured due to excessive physical activity	8 (2.6)	1–2	9	2 (25.0)
Factor 3: Burn and fire	35 (11.4)	1–3	46	6 (17.1)
Burned by hot water	15 (4.9)	1–2	16	3 (20.0)
Burned by hot objects (e.g., pot)	26 (8.4)	1–3	29	4 (15.4)
Fire or electrical accidents	1 (0.3)	1	1	1 (100.0)
Other	25 (8.1)	1–30	73	8 (32.0)
Factor 4: Suffocation/swallowing disorder	5 (1.6)	1–30	43	-
Factor 5: Medication-related incident	4 (1.3)	1–2	5	2 (50.0)
Factor 6: Dehydration	5 (1.6)	1	5	1 (20.0)
Factor 7: Heat-related illness (e.g., heatwave, heatstroke)	15 (4.9)	1–3	18	5 (33.0)
Factor 8: Cold-related illness (e.g., cold wave, frostbite, hypothermia)	2 (0.7)	1	2	-

^a^ Proportion of individuals with injury in the household in the past year among all subjects (n = 309). ^b^ Proportion of individuals who received hospital treatment (e.g., medication, physical therapy, surgery) due to injury.

**Table 3:** Risk factors for falls, cuts, and collisions (n = 309).

Characteristics	Categories	n (%)	Falls		Cuts and Collisions	
			OR [95% CI]	*p*	OR [95% CI]	*p*
Number of chronic diseases	0	59 (19.1)	1		1	
	1	137 (44.3)	0.78 [0.27–2.25]	0.639	0.62 [0.25–1.53]	0.302
	≥2	113 (36.6)	0.84 [0.25–2.82]	0.781	0.79 [0.28–2.21]	0.647
Number of medications	0	57 (18.4)	1		1	
	1	93 (30.1)	2.77 [0.85–9.02]	0.092	0.91 [0.34–2.39]	0.840
	≥2	159 (51.5)	3.59 [1.06–12.18]	0.041	1.30 [0.50–3.37]	0.586
Subjective health status	Unhealthy	100 (32.4)	1		1	
	Average	148 (47.9)	0.43 [0.19–0.99]	0.046	0.57 [0.26–1.25]	0.158
	Healthy	61 (19.7)	0.67 [0.23–1.96]	0.462	0.58 [0.20–1.67]	0.309
Sleep satisfaction	Dissatisfied	87 (28.2)	1		1	
	Average	112 (36.2)	0.96 [0.40–2.29]	0.931	1.17 [0.51–2.69]	0.706
	Satisfied	110 (35.6)	0.70 [0.27–1.79]	0.459	1.15 [0.47–2.80]	0.762
Frailty	Robust	125 (40.4)	1		1	
	Prefrail	143 (46.3)	1.10 [0.48–2.52]	0.820	0.66 [0.31–1.38]	0.269
	Frail	41 (13.3)	0.97 [0.26–3.64]	0.959	0.16 [0.04–0.67]	0.012
Perceived physical function	Vision impairment	158 (51.1)	0.44 [0.21–0.92]	0.030	2.15 [1.07–4.33]	0.032
	Hearing impairment	114 (36.9)	1.03 [0.47–2.28]	0.942	0.47 [0.22–0.99]	0.046
	Numbness in hands and feet	85 (27.5)	1.03 [0.44–2.45]	0.943	3.13 [1.39–7.07]	0.006
	Tremors in hands and feet	56 (18.1)	0.98 [0.34–2.84]	0.975	0.46 [0.16–1.29]	0.139
	Dizziness	82 (26.5)	4.37 [1.90–10.05]	0.001	0.92 [0.39–2.17]	0.852
Mobility aids	No	265 (85.8)	1	0.115	1	0.718
	Yes	44 (14.2)	2.35 [0.81–6.80]		0.82 [0.28–2.39]	
Depression	No	283 (91.6)	1	0.946	1	0.317
	Yes	26 (8.4)	1.04 [0.34–3.22]		0.54 [0.16–1.81]	
Outdoor falls	No	256 (82.9)	1	<0.001	-	
	Yes	53 (17.2)	8.36 [3.52–19.82]			
Indoor falls	No	222 (71.8)	-		1	<0.001
	Yes	87 (28.2)	-		6.29 [3.03–13.02]	
Indoor cuts/collisions	No	226 (73.0)	1	<0.001	-	
	Yes	83 (27.0)	6.20 [2.85–13.48]			
Indoor burns/fire	No	274 (88.6)	1	0.465	1	<0.001
	Yes	35 (11.4)	1.48 [0.52–4.24]		8.50 [3.31–21.85]	
Indoors, other injuries *	No	284 (91.9)	1	0.082	1	0.340
	Yes	25 (8.1)	2.85 [0.88–9.24]		1.72 [0.56–5.27]	

Multivariate logistic regression analysis adjusted for age and sex. * Suffocation/swallowing disorder, medication-related accidents, dehydration, and heat/cold-related illness. OR: odds ratio, CI: confidence interval.

## Data Availability

The datasets used and/or analyzed during the current study are available from the corresponding author upon request.
